# Overweight and Obese Adolescent Girls: The Importance of Promoting Sensible Eating and Activity Behaviors from the Start of the Adolescent Period

**DOI:** 10.3390/ijerph120202306

**Published:** 2015-02-17

**Authors:** Alwyn S. Todd, Steven J. Street, Jenny Ziviani, Nuala M. Byrne, Andrew P. Hills

**Affiliations:** 1 Centre for Nutrition and Exercise, Mater Research Institute, The University of Queensland, Brisbane 4101, Australia; E-Mails: alwyn.todd@mater.org.au (A.S.T.); steven.street@mater.uq.edu.au (S.J.S.); 2 Department of Nutrition and Dietetics, Mater Health Services, Brisbane 4101, Australia; 3 School of Allied Health Sciences, Griffith University, Gold Coast 4215, Australia; 4 Faculty of Health Sciences and Medicine, Bond University, Gold Coast 4226, Australia; E-Mail: nbyrne@bond.edu.au; 5 Children’s Health Queensland, Queensland Health, Brisbane 4000, Australia; E-Mail: j.ziviani@uq.edu.au; 6 School of Health and Rehabilitation Sciences, The University of Queensland, Brisbane 4067, Australia; 7 Bond Institute of Health and Sport, Bond University, Gold Coast 4226, Australia

**Keywords:** adolescence, girls, growth, nutrition, obesity, physical activity, pregnancy

## Abstract

The adolescent period is associated with changes in eating and activity behaviors in girls. Less reliance on parental provision and choice of food, coupled with a decrease in participation in physical activity and sport, can create an energy imbalance, predisposing to weight gain. Physiological alterations to body composition, reduction in insulin sensitivity, and psychological adjustments may further amplify the risk of becoming overweight and maintaining an unhealthy level of body fat into childbearing years. During pregnancy excess body fat is a risk factor for poor pregnancy outcomes and may predispose an infant to a lifelong heightened risk of being overweight and developing chronic disease. Interventions aimed at preventing the accumulation of body fat in adolescent girls and young women may have far reaching impact and be critically important in reducing intergenerational weight gain. Lifestyle interventions in adolescence have the potential to modify adult obesity risk by switching at-risk individuals from a high to lower obesity risk trajectory. This paper discusses multiple approaches to assist at-risk individuals reduce obesity risk. A key focus is placed on engagement in food preparation and choice, and opportunities for physical activity and sport. Support, education, and opportunity at home and at school, are often associated with the success of lifestyle interventions, and may enable adolescents to make positive choices, and engage in health promoting behaviors during adolescence and childbearing years.

## 1. Introduction: Adolescence, a Critical Period to Modify Obesity Risk

Understanding the links between female adolescent development, weight gain, subsequent maternal obesity, and adverse pregnancy outcomes is critical if we are to improve the health of future generations. Excess weight gain during adolescence often persists into adult life and is compounded during childbearing years. Adolescence is a “high-risk period” for weight gain, characterized by critical changes in body composition, insulin sensitivity, eating and activity behaviors, and psychological adjustments [1,2,3]. Excess weight gain during this key transitional period places an adolescent girl at increased risk of maintaining unhealthy levels of body fat in childbearing years. As adolescence is a time of developmental plasticity [4,5] in which lifelong habits can become established, lifestyle interventions during this period may have a significant influence on lifelong health. Specifically, the promotion of sensible eating and physical activity during adolescence may modify an adolescent’s risk of adult obesity [1,6].

Obesity is a major and complex health issue throughout the developed world, with an estimated three-fifths of Australian adults overweight or obese [7]. Treatment of obesity is complex and costly, and can involve various weight management options including behavioral, pharmacologic, and surgical interventions. The adolescent period represents an important window of opportunity for lifestyle intervention to prevent and manage longer term body fat accumulation [1,6].

Participation in physical activity and sport, both in and out of school, decreases during adolescence, particularly for girls [8]. Diet also undergoes significant changes [9] in many individuals consistent with the development of greater autonomy over food intake. Changes in body size, shape, and composition during the pubertal and adolescent periods may trigger body dissatisfaction and unhealthy eating and weight control practices, such as skipping meals, severely restricting intake of carbohydrate, protein or dairy foods, laxative use and smoking [10,11]. Promoting healthy lifestyle behaviors, such as physical activity and exercise, along with healthy dietary choices, could modify obesity risk and improve the health of future generations [12]. The identification of effective methods of intervention at this time may reduce the cycle of intergenerational weight gain, as offspring born to healthy-weight mothers have less risk of becoming overweight and developing chronic diseases later in life [12]. 

Adolescence can also be a challenging time for individuals, their families, and health professionals involved in their support and care. It is also a time that focused interventions may have a far-reaching impact in terms of future disease risk and fetal health. The aim of this review is to discuss the important physiological, behavioral, and psychosocial changes that occur during adolescence that may increase the risk of carrying excess weight into childbearing years, and to consider promising approaches to assist at-risk individuals.

## 2. Physiological, Behavioral and Psychosocial Changes during Adolescence

### 2.1. Physiological Changes during Adolescence

The adolescent body undergoes reproductive maturation and physical growth during the transition to adulthood. Alterations to hormones, glucose metabolism, and insulin resistance occur along with changes in body shape and composition [2] at a speed that is often bewildering to the individuals in which this is happening. For many adolescents, becoming comfortable with their bodily changes is challenging [13,14], and they may experience feelings of awkwardness.

The body shape of the adolescent girl transforms from immature child to mature adult. Changes in shape are highly variable with normal growth and development complicated by early and late patterns of maturation. Typically, the average female matures 18 months to 2 years earlier than her male counterpart [2] and females accumulate a much greater percentage of fat, and less fat-free mass than males [15]. Early maturing girls can attain peak height and weight, and develop adult levels of adiposity and lean body mass, by approximately 12 years of age [12,16]. Body composition changes for all adolescent girls include alterations in both the quantity and distribution of body fat [17]. Typically, fat deposition aggregates around the hips [15,17] consistent with a wider pelvic structure and breasts, with physical alterations triggered by changing levels of estrogen, testosterone, and growth hormone, along with an increase in the number and size of adipocytes [18]. It is difficult to determine normal weight gain during this transitional period, however, as marked changes in body composition occur, and some adolescents have an increased risk of overweight and obesity [19,20,21]. Early onset of menarche is associated with greater risk of obesity [12,16,22,23] as is the case for pre-pubertal obesity [24,25]. In contrast, particularly for those who are physically active and consume a healthy diet, the adolescent period may present an opportunity to outgrow unhealthy excess pre-pubertal body fat. 

#### 2.1.1. Potential Implications of Excess Body Fat Accumulation in Adolescence

Excess body fat during adolescence contributes to a greater health risk in adolescence and adulthood, particularly in childbearing years when excess weight can lead to poor pregnancy outcomes [12]. Obese adolescents who remain obese as adults, also have an increased risk of developing chronic diseases later in life [26]. It is therefore of critical importance that this developmental transition is managed well by young girls, their parents, and health practitioners [27].

Adolescent growth has also been linked with a substantial reduction in insulin sensitivity that may be exaggerated in the presence of excess body fat accumulation [28,29,30,31]. An increase in growth hormone may be, at least in part, responsible for this reduction, but the exact pathway remains uncertain [1]. The reduction in insulin sensitivity is preceded by high levels of plasma insulin in adolescence, leading some researchers [32] to hypothesize that this may be a factor in the onset of menarche. Although a decrease in insulin sensitivity is a normal occurrence during adolescence [1], the maintenance of decreased insulin sensitivity into childbearing years is likely to be dependent on lifestyle factors and body composition. Decreased insulin sensitivity during childbearing years is associated with inappropriate gestational weight gain and poor pregnancy-related outcomes, as well as longer-term risk of chronic disease for both mother and child [33]. Sensible eating and physical activity through adolescence should increase insulin sensitivity and discourage excessive accumulation of body fat [34], thereby improving pregnancy outcomes. Ultimately, a successful intervention in adolescence that improves health may prevent the development of obesity, reduce the risk of gestational diabetes mellitus, pre-eclampsia, and back pain in pregnancy, and increase the chances of delivering a healthy infant [12,35].

#### 2.1.2. Measuring Physiological Changes in Adolescence

The body mass index (BMI) adjusted for age and sex (Adj-BMI) is an accepted ratio of weight to height in adolescence, correlates well with adiposity [36] and is preferred over weight-for-age or weight-for-height comparisons [36]. The Adj-BMI is determined by calculating BMI (weight/height^2^) and mapping the adolescent’s weight and height against an appropriate BMI chart adjusted for the age and sex. As BMI and patterns of growth differ between adolescents, clinical judgment is also required when an adolescent is close to the centile cut-offs that correspond to the “overweight” and “obese” classifications [36]. Adj-BMI > 85th percentile indicates an adolescent may be overweight, and >95–97th percentile (depending on the growth chart selected), indicates obesity. The specificity of BMI centiles may be reduced in the presence of a broader bone structure or increased muscle mass, the latter often observed in athletes. Growth spurts may also temporarily reduce BMI centiles, if growth precedes other aspects of development. The same chart should be used to track growth over time and monitor BMI increases or percentile crossing [36]. An alternative approach to monitoring relative weight in adolescence, the “waist-to-height ratio” (WHtR), does not rely on reference growth charts and correlates well with cardio-metabolic risk factors [37]. However, this approach is not widely used due to limited data on its accuracy [36].

### 2.2. Behavioral Changes during Adolescence

In recent decades, dramatic increases in the prevalence of childhood and adolescent overweight and obesity have occurred in tandem with reductions in habitual physical activity and exercise, and the availability of energy-dense foods. The growing years have traditionally been considered “critical” or “sensitive” periods when the risk of onset, complications or persistence of overweight and obesity is potentially increased [38]. Such periods include adolescence when changes in growth, development and maturation can be rapid. Similarly important transition periods are from high school to higher education, or from school into the workforce [39]. 

Eating, physical activity and sedentary behaviors have a long lasting impact on the physical health of adolescents [40]. These behaviors, in isolation or combination, may lead to an imbalance between energy intake and energy expenditure [41,42,43] and predispose an individual to underweight or overweight. Nutrient deficiencies can also develop if the nutrient density of foods consumed is poor [44]. In Westernized societies, a high energy intake (with a greater number of food items of poor nutrient density) and low level of physical activity (accompanied by a greater proportion of time being sedentary), are areas of concern for overweight and obesity in adolescents [36].

#### 2.2.1. Food Choice and Dietary Intake

Adolescence is also a time when food choice and dietary intake moves from being almost solely determined by parents/guardians, to one where adolescents take greater responsibility for their dietary choices. Consistent with greater control over dietary choice is the potential for changes in food preference [3] driven by lifestyle, hormonal, social or environmental alterations [3]. Adolescence is often associated with an increase in sleep duration which may impact eating routines and the regulation of food intake [45]. Hormonal changes may be implicated in a preference for salty, sweet, or high fat foods [46,47,48]. Dietary preferences of siblings and peers, television advertising and social marketing, can also influence food choices [49,50]. These changes, coupled with the ready availability of inexpensive foods and beverages high in calories and low in nutrients [51,52] can lead to replacement of healthy foods with those high in fat and sugar. Such dietary changes may alter energy balance, be less nutritious and limit the ability to meet micronutrient requirements [53]. 

#### 2.2.2. Body Image and Unhealthy Weight Control Behaviors

During adolescence females become more aware of their body size and shape, and observational research indicates that body satisfaction may decrease, especially in overweight girls [1,54]. For some, this can lead to dieting and unhealthy weight control behaviors that may continue into adult life. Stigma, bullying and discrimination related to body weight during adolescence can also increase the likelihood of adolescent girls developing an eating disorder or obesity.

A recent study of adolescent eating behaviors found that adolescent girls were less likely to diet, binge eat, and attempt to control their weight in unhealthy ways if their parents engaged in healthy eating rather than weight-focused conversations [55], highlighting the importance of family in managing adolescent eating habits. Focusing on conversations around healthy eating and being active instead of weight may enable adolescents to indirectly reduce adiposity without the focus being on their body size or shape, minimising the risk of developing an unhealthy body image or relationship with food.

#### 2.2.3. Sedentary Behaviors and Physical Activity

Physical activity is important for healthy growth and development across childhood and adolescence. Participation in regular physical activity is essential for normal motor development, including the acquisition of fundamental motor skills necessary for engagement in activities of daily living and sports-specific tasks [56,57]. The well-documented health benefits of physical activity for young people include improved body composition and the prevention of overweight and obesity; plus benefits for skeletal, cardiovascular and psychological health [58,59]. In contrast, low levels of physical activity and a predisposition to sedentary behaviors are consistent with an early exposure to adult-onset health conditions [60,61,62]. Sadly, too many children and adolescents, and young girls in particular, are not sufficiently active to maximize health benefits. Inappropriate levels of physical activity and poor eating habits are major determinants of obesity in childhood. If this scenario straddles the pubertal barrier into adolescence, a vicious cycle of limited physical activity experiences, sedentary behaviors and poor eating habits is perpetuated [63].

Sedentary time has been shown to increase in adolescence [64]. Greater sedentary time is associated with poorer metabolic health and may be linked to obesity development in adolescents [65,66,67]. Being less active may increase the risk of gaining excess body fat or reduce the likelihood of an overweight female adolescent outgrowing adiposity during the transition to adulthood. Many environmental factors may predispose adolescents to greater sedentary behaviors, such as increased television screen time [67,68], greater interest in personal communication platforms and the internet [68], less active modes of transportation to and from school and heightened awareness of personal security [69].

Participation in physical activity and sport is known to decrease in adolescent girls [70,71]. This decrease is thought to coincide with the onset of menarche. Girls who have been active children and enjoyed participating in sports commonly become less active adolescents. Research has shown common reasons for decreased participation in sport include negative experiences undertaking physical activity at school, a perceived lack of personal ability or perceived lack of sports ability acknowledgement from teachers and greater focus on other non-sport interests [71]. Negative experiences of undertaking physical activity at school may be related to the physical changes females experience with their bodies during adolescence [72] and the psychosocial aspects of these changes; such as embarrassment and uncomfortable feelings about the changes their bodies are undergoing, worrying about the appearance of their bodies in sports gear or the potential for clothing to become dislodged during physical activity, and not wanting to change in front of people.

Girls often partake in sports such as dancing and gymnastics that have a competitive time frame after which many individuals cease to participate if they are not competitive at a regional or national level. Giving up this type of sport is often not mandated by sports coaches or organizations, but may be influenced by sports coaches focusing more on individuals whom they deem to be more competitive leaving other individuals feeling there is little reward to continuing to participate. Incidental and lunch time physical activity and sports may also decrease as adolescent girls become more interested in the social aspects of school and life, and more aware of male adolescents. Lunch and after school time may be spent socializing with friends, rather than playing games on the school sports field.

Environmental changes may also impact a female’s sport and activity levels. Urbanization, technology such as cell phones and the internet, and increased use of automated transport may all be factors which impact the amount of physical activity and exercise undertaken by adolescent girls. Community access to recreation facilities, transition from primary to secondary school and the cost of sports and recreation activities may also impact a parent or guardians decision to encourage their children to continue activities through the adolescent year [73].

### 2.3. Psychosocial Changes during Adolescence

In addition to physical changes, psychological and cognitive maturation, and shifts in understanding social roles are characteristic of the transition to adolescence. Cognitive fluctuations in IQ and executive function reflect neuronal reorganization and re-wiring, a protracted process that continues into young adulthood [74]. A psychological mechanism through which self-regulation modifies obesity risk may be the formation of identity during adolescence.

#### 2.3.1. Executive Function and Self-Regulatory Skills

Executive function underpins behavioral self-regulation. Therefore, it is not surprising that difficulties in executive function in adolescence are associated with obesity [75], dietary restraint and disinhibited eating in obese adolescents [76]. Thus, it is possible that bolstering self-regulatory capacity leading into adolescence may be prophylactic for obesity, a notion for which there is some empirical support [77,78,79]. It is certainly the case that children raised in physically, socially, and intellectually supportive environments tend to have better executive function [80]. Furthermore, parenting style, a critical component in the provision of such an environment, may also contribute independently to obesity risk. For example, children of authoritarian (low acceptance—highly controlling) and disengaged (low acceptance—low controlling) parents are at a greater risk of obesity compared to authoritative parents (high acceptance—high controlling) in the transition from adolescence to young adulthood [81]. These data suggest the provision of a coherent and supportive framework with clearly defined boundaries in the family setting is crucial to the development of self-regulatory skills in adolescence, and that one manifestation of these skills is self-regulation of a healthy diet. 

#### 2.3.2. Identity Formation and Cultivation of Healthy Behaviors

Development of self-regulatory capacity is associated with identity development [82]. Although current research is limited, there is good reason to think a relationship between these factors contributes to adolescents identifying as “healthy eaters” or as “exercisers”. According to Identity Theory [83], identity adoption and identity-congruent behavior are highly correlated, a hypothesis that has empirical support [84,85]. Familial support appears to be crucial during adolescence in fostering this sense of identity. Complete independence is still a future prospect for most adolescents and guidance received from parents can influence the way adolescents perceive themselves [86]. However, conflict between parental ideals, desire to be accepted by peers, and an adolescent’s sense of their emerging identity can create intra and inter personal tension and conflict that may exacerbate behaviors associated with health risks [86]. Thus, family and peer relationships are a nexus around which psychological factors that predispose to obesity risk, such as identity development, may be moderated particularly as adolescents move into adulthood. 

**Figure 1:**
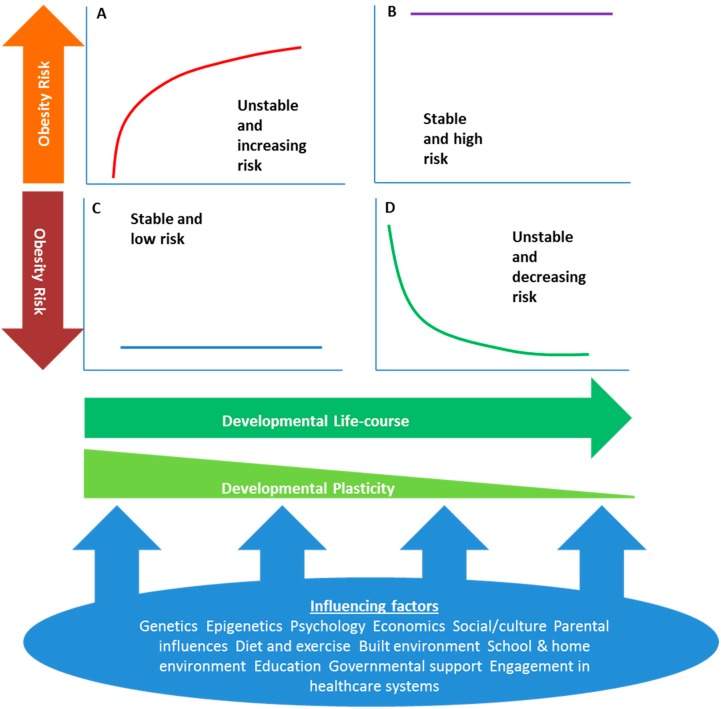
Obesity risk trajectories for adolescents: (**A**) Normal weight individual, BMI increasing, obesity risk increasing; (**B**) Overweight individual with stable BMI, high obesity risk; (**C**) Normal weight individual with stable BMI, low obesity risk; (**D**) Overweight individual, BMI decreasing, obesity risk decreasing. Obesity risk may be improved by lifestyle interventions that assist in switching individuals from trajectories A and B to trajectory D.

Cultivating healthy behaviors during adolescence may increase the potential for healthy habits in adulthood. For example, healthy dietary habits modeled in the home have been shown to persist when children move out [87]. This relationship between habit formation and the early establishment of healthy behaviors has been and remains an active area of research [88]. Furthermore, these habits are likely to be bound to an individual’s identity [85] particularly if identity strength is high [89]. Taken together, the research suggests that family settings are highly influential for the formation of adolescent identity. This identity is likely to be congruent with a suite of behaviors, which if cultivated, are likely to become habits. Continuity between healthy behavior in adolescence and adulthood has received empirical support suggesting healthy habits are resilient to the turbulence of adolescence. Extrapolating these findings along the developmental trajectory, and in light of evidence from research into the developmental origins of health and disease (DOHaD) hypothesis, healthy habits may be trans-generational, with carry-over benefits accruing to subsequent generations through the transmission of healthy habits to offspring [12]. Thus, disclosing differential levels of risk in adolescent girls (see Figure 1), and intervening to assist in the development of positive and healthy identity formation, is an essential strategy with the potential to improve the future health of young mothers and babies.

## 3. Approaches to Assist At-Risk Individuals Reduce Obesity Risk

Interventions in adolescence have the potential to modify adult obesity risk by switching at-risk individuals from a high to lower obesity risk trajectory (Figure 1) [12,90,91]. Multicomponent lifestyle interventions including a combination of diet, physical activity, and other behavioral strategies, are likely to be the most successful [36]. Interventions in early adolescence may be more effective than later interventions. As many women struggle to implement healthy eating and lifestyle behaviors when trying to conceive or during pregnancy [92], there may be merit in encouraging adolescents to establish healthier behaviors so key concepts are more familiar, and easier to implement during pregnancy [12]. Long-term prospective cohort studies are also warranted to identify if such interventions during adolescence can help girls establish healthy eating and lifestyle behaviors and enable the achievement of a more optimal health status during childbearing years [12].

### 3.1. Individual vs. Family Approaches

Evidence suggests there should be a focus on the health behaviors of the whole family rather than the weight of an individual child or adolescent [36,93,94,95]. This is consistent with a supportive and collaborative home environment assisting in the prevention of obesity and the adoption of healthy eating and activity behaviors in adolescent girls [96]. Providing opportunities to cook and prepare food, and undertake a wide range of outdoor activities, may help to create a more stable, happy and healthy family [97,98], and develop healthy behaviors to carry into young adulthood and later life. The stage of maturity, age, and preferences of an adolescent girl should be considered when deciding what types of activities might be appropriate, and the level of family involvement required. Some adolescents may prefer to join sports groups, or undertake activities with friends, rather than spend time with family members. However, seeing other family members engaging in healthy lifestyle behaviors may help the adolescent feel supported rather than isolated or singled out as individuals needing to make more effort than the rest of the household [25]. Changes in parental behavior and weight have also been linked to successful weight loss in adolescents [99]. This is consistent with the notion of the value of family- rather than an individual-based approaches to behavior change [99]. General practitioners can also play an important role in healthy development, and should regularly engage in conversations around healthy eating and physical activity with adolescents and their parents [100].

In summary, evidence for supportive families and the link between parental-child discussions around weight and dieting or eating disorders [55], suggests family-centered behavior interventions focusing on healthy eating and physical activity can positively influence adolescent weight status, and minimize the risk of developing an unhealthy relationship with food, or poor body image. Focus groups conducted with adolescent girls have identified that both girls and their parents prefer health focused behavior change strategies as opposed to focusing specifically on the adolescent’s weight [101]. 

### 3.2. Nutrition

Sensible eating should be encouraged in adolescence to ensure optimal health, while dieting to lose weight should be avoided as this may disrupt eating patterns leading girls to restrict certain foods and eat in response to emotional rather than hunger cues [102]. A female adolescent requires adequate energy (kilojoules/calories) and nutrients to enable normal growth and development [53]. To meet nutritional requirements and avoid excess weight gain, a healthy dietary pattern consisting of fruit, vegetables, whole grains, lean meat, or alternative protein sources and dairy products, needs to be consumed; high fat, high sugar foods should be minimized [36,53]. It is especially important that inactive adolescents, those undertaking less physical activity than recommended, avoid high fat, high sugar foods. In adolescents who undertake large amounts of physical activity, energy requirements will be higher, and such individuals may need to increase their intake of healthy food accordingly. Provision of adequate calories for growth can be monitored by tracking physical changes and body composition. To minimize unhealthy weight gain, a reduction in calories and/or an increase in physical activity may be required [36]. Any reduction in energy intake should start with minimizing high fat, high sugar foods (such as convenience and takeaway foods, cakes and sweets, packaged snack foods, and sweetened beverages) and increasing intake of fruit, vegetables, whole grains, and low-fat dairy products.

Adolescents require a balanced intake of protein, fat, and carbohydrates [53] and overconsumption of one or more macronutrients may contribute to weight gain. In Australia, energy intake in 10–15 year-old girls increased by 11% between 1985 and 1995 (+900 kilojoules per day) [103]. Australia also now has one of the highest adolescent obesity rates in the world [104]. In order to normalise energy intake, prevent weight gain, and optimise nutrition for chronic disease prevention, complex food modelling was undertaken by the Australian Health and Medical Research Council in 2013 [105], to identify the key constituents of dietary patterns associated with decreased morbidity and mortality, such as the Mediterranean diet [106]. As dietary patterns differ amongst the numerous cultures within Australia and other western countries, this approach enables the promotion of healthful eating patterns within many different cultural food practices [53]. These guidelines suggest that adolescents and young adults may benefit from a balanced diet that has 15%–25% of dietary energy from protein, 25%–35% of dietary energy from fat and 45%–65% of dietary energy from carbohydrate. This macronutrient profile enables a high diet quality score, similar to that observed in a Mediterranean diet, and the inclusion of sufficient amounts of vegetables, fruit, whole grains, poultry, fish, and reduced fat dairy products to meet vitamin and mineral requirements [53]. Protein recommendations can easily be achieved by including protein-rich foods such as lean meat, fish, eggs, or legumes at lunch and dinner, and low-fat dairy products with meals or as drinks and snacks [53]. To meet the dietary fat recommendations, only a small amount of added fat may be required (two-three teaspoons (tsp) per day) as fat is present in many other foods. Added fats should be predominantly mono- and poly-unsaturated, and this amount should include oil used in cooking, spreads such as butter, margarine, and peanut butter (two tsp is equivalent to one tsp fat), and fat from nuts, seeds, and avocado (four almonds, two walnuts, or two slices of avocado are each equivalent to one tsp fat) [53]. Carbohydrate can be achieved by consuming cereal or toast at breakfast, and lunch and dinner meals including a fist-sized serve of either rice, pasta, couscous, quinoa, or wholegrain bread [53]. A practical way to meet food group recommendations is to think about the foods served up for lunch and dinner. Carbohydrate-rich foods should comprise ⅓ of a meal, protein the other ⅓, and vegetables the remainder. Plate size is also a consideration, and a helpful strategy for parents may be to purchase smaller dinner plates [107]. It is also important to ensure adequate fiber and calcium intake, as these are not only essential nutrients for healthy gut function and bone metabolism, but also assist with the maintenance of a healthy weight [26]. Choosing wholegrain carbohydrates and consuming five serves of vegetables (½ cup cooked vegetables or one cup salad is equivalent to one serve), and two of fruit per day will ensure adequate fiber. Dairy is the main source of dietary calcium, and female adolescents require 3½ serves per day (one cup of milk, ¾ cup of yogurt, or two slices of cheese are equivalent to one serve) [53].

Evidence from clinical studies shows that protein at breakfast may help adolescent girls maintain a healthy body weight [108] by contributing to less hunger and an increased feeling of “fullness” throughout the day [108]. Feeling of fullness was greatest for those who consumed a high protein breakfast (35 g of protein), and girls who consumed a normal protein, high carbohydrate breakfast (13 g of protein), reported increased “fullness” compared to the breakfast-skipping arm. These data support the consumption of high protein foods such as egg and meat at breakfast to assist adolescent girls develop a healthy eating pattern that increases satiety, decreases appetite, and reduces snacking on high fat foods. 

Diet quality during adolescence may also have major implications for body weight, body composition, and chronic disease during adult life including the development of obesity, metabolic syndrome, and co-morbidities such as diabetes, hypertension, and cardiovascular disease [1,109]. A diet high in energy and low in nutrients during adolescence may lead to higher levels of body fat, especially in the presence of low levels of physical activity [110,111]. As an adolescent’s body undergoes physical and metabolic changes, including a period of decreased insulin sensitivity, healthy eating is paramount to ensure excess body fat does not accumulate, aggravate insulin resistance, and predispose to a sub-optimal environment for fetal development, should the woman become pregnant [28,112]. Appropriate healthy eating and exercise interventions in children and adolescents can lead to weight loss and an improvement in triglyceride and low density lipoprotein levels [110] plus favorable changes in high density lipoprotein levels, fasting glucose, and insulin [110]. Such changes may favorably influence the weight, body composition, and metabolic profile of adolescent girls, predisposing them to a healthier adult weight and body composition and improving their chances of giving birth to healthy offspring.

During adolescence, many girls increase their self-selection of food rather than eating all meals provided in the home. Whenever possible, parents should engage adolescent girls in food shopping, cooking, and meal planning, to maximize their development of both nutritional knowledge and food skills. The establishment of healthy eating routines in the home, including sitting down to meals as a family and turning the television off at meal times is very important [36] with girls being more likely to connect eating with their nutritional requirement for food, and recognize the sensations of hunger and satiety. Family meals have also been associated with higher diet quality scores and the development of a supportive family environment [26]. 

### 3.3. Physical Activity

A coordinated approach to the prevention and/or management of overweight and obesity through increased physical activity in adolescence would more likely influence the knowledge, attitudes and behaviors of adolescents as young parents. Healthier pregnancies in turn are consistent with improved health outcomes for offspring. Infants and young children depend on responsible adults for guidance therefore all adults should be role models for acceptable eating and activity behaviors commencing from a young age.

In relation to the current obesity epidemic, Olshansky *et al.* [113] have suggested that “the youth of today may, on average, live less healthy and possibly even shorter lives than their parents”. To ameliorate this problem, and maximize the benefits from physical activity and exercise in the context of body composition and weight management, greater emphasis is needed to match public health physical activity promotion strategies with individual needs, including optimizing exercise prescription. Increasing knowledge and understanding of benefits, plus encouraging enjoyment in physical activity, is consistent with increased individual responsibility for activity participation and health more broadly. An optimal scenario would be to prevent the progression or development of overweight and obesity in adolescents ahead of the adult years [61,114,115].

Critical periods of growth and development may be the most important times for increasing activity opportunities, particularly as activity levels decline rapidly in many adolescent girls. As physical activity participation likely tracks across the growing years and into adulthood [116], exposure to active play opportunities should commence early in life and physical activity should continue to evolve, and be promoted throughout the growing years. As the decline in adolescent physical activity is greater in girls interventions may be more effective if they specifically target girls alone, and there is some data to support this notion [117]. Physical activity interventions targeting the early adolescent period, delivered in school based settings, and addressing both physical activity and sedentary behaviors have also been found to have greater trends towards success in adolescent girls [117]. 

There are a number of published reports of school-based trials showing promising results for increasing physical activity in adolescent girls. A multi-center cluster randomized controlled trial of school based physical activity for 90 min from 4–5.30 pm on two weekdays and 150 min on a Saturday, *vs.* no intervention reported a significant reduction in adiposity and improvements in cardiometabolic health for Spanish girls aged 8–10 years [118]. A study in the United States reported that hours of physical education were positively linked to physical education enjoyment, and physical activity score, and physical education enjoyment was positively linked to use of active transport to school [119]. A Finnish study reported physical activity at recess was correlated with more positive peer relationships and peer support [120]. These studies demonstrate that school based physical activity opportunities and interventions can positively influence adolescent girl’s relationship with physical activity and may also enhance psychological wellbeing. An additional advantage of school-based interventions is the reduced emphasis on individual accountability, and greater emphasis on the roles of schools to provide appropriate opportunities and create supportive environments for physical activity.

As neighborhood environment can also encourage or discourage physical activity this may be a target area to passively encourage greater physical activity of resident adolescents. Access to safe environments with interesting places and things that adolescent girls like has been correlated with increased physical activity levels [121]. Targeting lower income areas that have fewer parks and facilities for redevelopment investment may assist in providing a safer and more enjoyable environment for adolescent girls.

### 3.4. Sleep Duration

Hours of sleep during adolescence have been inversely correlated with weight gain, with stronger associations observed in those who are already significantly overweight. A study of U.S. adolescents, between grades nine and twelve, found those who slept for longer each night gained less weight than those with a similar BMI. An increase in sleep from 7.5 to 10 h per day was associated with a 4% reduction in the prevalence of overweight/obesity [122]. Increasing sleep duration may favourably influence energy metabolism by altering the secretion of leptin and ghrelin [45], previous studies have shown lower levels of leptin and higher levels of ghrelin with increasing sleep duration, and correlated outcomes with BMI [45]. The effect on BMI may be mediated via a reduction in appetite and food intake [123] associated with alterations in appetite hormones. Other research has not shown changes in leptin and ghrelin with sleep restriction, but instead an increase in reward driven food intake, so it is possible that the correlations of shorter sleep duration with increasing weight may be due to changes in brain signalling and food intake [124] rather than actual duration of sleep *per se*.

### 3.5. Promising Interventions

It has also been hypothesized that adolescence may be an appropriate time to educate adolescents about DOHaD concepts [12]. Bay *et al.* utilized a school-based educational intervention to teach DOHaD concepts to 11–14 year olds and assess if this initiative could improve understanding of DOHaD concepts and facilitate discussion of these concepts within families [12]. The program operated across nine schools in New Zealand, and found that the coursework increased knowledge of the link between dietary intake in pregnancy and health of offspring in adulthood and also stimulated adolescents to engage in discussions with their families about diet and lifestyle, and the DOHaD concepts they learned. 

Good-quality evidence demonstrates that women who become pregnant at a healthy weight have a lower risk of metabolic complications and assisted deliveries, and their infants have a reduced risk of macrosomia, structural birth defects, perinatal death, obesity and chronic disease in childhood and adolescence [33]. Diet and lifestyle interventions that tailor programs for activity and provide education and support with nutrition can reduce weight in childhood and adolescence, and favorably influence the weight, body composition and metabolic profile of adolescent girls [110]. Therefore, interventions that target healthy lifestyle behaviors in adolescence hold promise for reducing the risk of pregnancy-related complications [12]. To date, no published studies have intervened in adolescents and prospectively followed participants into the childbearing years to examine outcomes. Studies are needed that implement multicomponent lifestyle behavior interventions in adolescence and prospectively follow participants to determine if the intervention resulted in an improvement in infant health outcomes.

Adolescent girls who develop severe obesity and co-morbidities and struggle to reduce body fat using lifestyle and educational approaches may benefit from specialist pre-conception interventions [36] such as a very low calorie diet, or as a last resort, bariatric surgery. Bariatric surgery interventions have been found to reduce the risk of gestational diabetes mellitus, preeclampsia, and hypertension during pregnancy, and infant risk of macrosomia in women who were previously obese [125]. Such specialist approaches should only be used post-puberty, when the requirements for growth and development would not be compromised [36]. Interventions require specialist weight management services including medical consultant, dietitian, exercise physiologist and psychologist. 

### 3.6. Sustainability

Sustainability is a key part of any intervention or strategy to improve population health [126]. Lifestyle interventions must be achievable and sustainable over the longer term if a long term health benefit is to be observed. Some previous school based interventions focusing on implementing healthy eating and lifestyle concepts have been successful in achieving long term change towards a healthier food and physical activity environment [126]. One key to sustainability is acceptance and “buy in” from the target population. To achieve sustainability adolescent girls should be encouraged to engage and have input into planned changes within schools to offer greater opportunities for physical activity, to ensure types of physical activity adolescent girls enjoy are made available. Girls could also be encouraged to take an active role in improving the food environment within schools, providing input into the types of food dishes they enjoy and would like to be available in the school cafeteria, and input into the way that vegetables are prepared to make them more enjoyable and increase consumption. Having adequate opportunities within schools for students to develop cooking and gardening skills may also assist in raising awareness and interest in food preparation and healthy eating.

Interventions should focus on behavior change and the longer term, creating sustainable healthy eating changes and improving the environment to enable more opportunities for physical activity, not just at the present time, but also into the future. The greater the longer term focus on small manageable longer term behaviour change and environmental improvement, opposed to a short term diet and exercise regimes, the greater the likelihood of sustainability.

## 4. Future Directions

The link between adolescent obesity, maternal obesity, and pregnancy-related health outcomes is still to be firmly established. It is likely that the ideal management will be prevention, specifically prevention of the transition of weight gain in adolescence to overweight in young adulthood and pregnancy. The rise in obesity in pregnancy, childhood, and adolescence, coupled with the associated risk of chronic conditions such as type 2 diabetes mellitus and cardiovascular disease, is setting the scene for a significantly unhealthier population in the future, ever-increasing and unmanageable health care costs, and potentially, premature death [127]. Adolescent girls should be a priority population for strategic intervention as adolescence is a “window of opportunity” to reduce the incidence of being overweight in pregnancy, and the burden of lifetime disease for a future mother and her children. Furthermore, as many adolescent girls will become mothers and play a central role in food provision, access to physical activity and sports, teaching cooking skills, and educating children about nutrition, should significantly influence the risk of obesity and associated conditions.

Adolescent interventions to improve lifestyle behaviors should be designed to maximize impact and minimize harm with a focus on lifestyle and behavior change, rather than weight as an outcome. This is important to reduce the risk of negative self-esteem and unhealthy relationships with diet and exercise. There may also be a role for interventions delivered by social media (Facebook, Twitter, smartphones) in the provision of programs that focus on lifestyle change [128,129,130]. Multicomponent interventions are likely to have the greatest impact, but will require significant planning and the involvement of researchers, doctors, psychologists, dietitians, and exercise physiologists. Individual clinicians treating and advising adolescents should encourage the development of a healthy diet, increased activity and healthy sleep behaviors. 

## 5. Conclusions 

Provision of opportunities to adopt healthy eating and activity behaviors in adolescence may reduce obesity risk and assist girls achieve a healthier body composition before they transition to childbearing years. Engagement in food preparation and choice, and education around healthy eating can assist with the adoption of healthier eating habits, and may have an impact on an individual’s food choice during pregnancy, and later life. Providing opportunities for engagement in physical activity and sport, to an individual and as a family, will decrease sedentary time, optimise metabolic health and reduce the risk of weight gain. Education and opportunities provided concurrently at home and school, involving family, and encouraging the development of sibling and peer support for healthy eating and activity behaviors are likely to have the greatest impact. Reducing the obesity risk trajectory of adolescent girls has the potential to improve pregnancy outcomes and optimise the health of future generations. 

**Author Contributions**

Alwyn S. Todd, Steven J. Street and Andrew P. Hills work together. Alwyn S. Todd prepared the sections on nutrition and drafted the article with input from other authors. Andrew P. Hills provided the overall concept for the paper, and together with Nuala M. Byrne prepared the sections on physical activity. Steven S Street prepared the section on psychosocial changes, and developed the concept of the obesity risk trajectory (Figure 1). Jenny Ziviani contributed to content on obesity risk, physiological changes during adolescence, and strategies to assist at risk individuals. 

**Conflicts of Interest**

The authors declare no conflict of interest. 

## References

[1] Alberga A.S., Sigal R.J., Goldfield G., Prud'homme D., Kenny G.P. (2012). Overweight and obese teenagers: Why is adolescence a critical period?. Pediatr. Obes..

[2] Hills A.P., Byrne N.M. (2010). An overview of physical growth and maturation. Med. Sport Sci..

[3] Neumark-Sztainer D., Story M., Perry C., Casey M.A. (1999). Factors influencing food choices of adolescents: Findings from focus-group discussions with adolescents. J. Amer. Diet. Assn..

[4] Hochberg Z., Belsky J. (2013). Evo-devo of human adolescence: Beyond disease models of early puberty. BMC Med..

[5] Hochberg Z. (2011). Developmental plasticity in child growth and maturation. Front. Endocrinol..

[6] Hills A.P., Andersen L.B., Byrne N.M. (2011). Physical activity and obesity in children. Brit. J. Sport. Med..

[7] Australian Institute of Health and Welfare Overweight and Obesity. http://www.aihw.gov.au/overweight-and-obesity/.

[8] Molnar D., Livingstone B. (2000). Physical activity in relation to overweight and obesity in children and adolescents. Eur. J. Pediatr..

[9] Demory-Luce D., Morales M., Nicklas T., Baranowski T., Zakeri I., Berenson G. (2004). Changes in food group consumption patterns from childhood to young adulthood: The Bogalusa heart study. J. Amer. Diet. Assn..

[10] Sonneville K.R., Calzo J.P., Horton N.J., Haines J., Austin S.B., Field A.E. (2012). Body satisfaction, weight gain and binge eating among overweight adolescent girls. Int. J. Obes..

[11] Vander Wal J.S. (2012). The relationship between body mass index and unhealthy weight control behaviors among adolescents: The role of family and peer social support. Econ. Hum. Biol..

[12] Bay J.L., Mora H.A., Sloboda D.M., Morton S.M., Vickers M.H., Gluckman P.D. (2012). Adolescent understanding of DOHaD concepts: A school-based intervention to support knowledge translation and behaviour change. J. Dev. Orig. Health Dis..

[13] Murray K., Rieger E., Byrne D. (2013). A longitudinal investigation of the mediating role of self-esteem and body importance in the relationship between stress and body dissatisfaction in adolescent females and males. Body Image.

[14] Chung R.J., Sherman L., Goodman E., Bickham D.S., Rich M. (2013). Exploring the perspectives of obese adolescent girls. Qual. Health Res..

[15] Pietrobelli A., Boner A.L., Tato L. (2005). Adipose tissue and metabolic effects: New insight into measurements. Int. J. Obes..

[16] Lloyd T., Chinchilli V.M., Eggli D.F., Rollings N., Kulin H.E. (1998). Body composition development of adolescent white females: The Penn State young womenʼs health study. Arch. Pediatr. Adolesc. Med..

[17] De Ridder C.M., Thijssen J.H., Bruning P.F., van den Brande J.L., Zonderland M.L., Erich W.B. (1992). Body fat mass, body fat distribution, and pubertal development: A longitudinal study of physical and hormonal sexual maturation of girls. J. Clin. Endocrinol. Metab..

[18] Janesick A., Blumberg B., Diamanti-Kandarakis E., Gore A.C. (2012). Adipocytes as target cells for endocrine disruption. Endocrine Disruptors and Puberty.

[19] Limbers C.A., Young D., Grimes G.R. (2014). Dietary, physical activity, and sedentary behaviors associated with percent body fat in rural Hispanic youth. J. Pediatr. Health Care.

[20] Malina R.M., Huang Y.C., Brown K.H. (1995). Subcutaneous adipose tissue distribution in adolescent girls of four ethnic groups. Int. J. Obes. Relat. Metab. Disord..

[21] Rolland-Cachera M.F., Bellisle F., Deheeger M., Pequignot F., Sempe M. (1990). Influence of body fat distribution during childhood on body fat distribution in adulthood: A two-decade follow-up study. Int. J. Obes..

[22] Wattigney W.A., Srinivasan S.R., Chen W., Greenlund K.J., Berenson G.S. (1999). Secular trend of earlier onset of menarche with increasing obesity in black and white girls: The Bogalusa heart study. Ethn. Dis..

[23] Freedman D.S., Khan L.K., Serdula M.K., Dietz W.H., Srinivasan S.R., Berenson G.S. (2003). The relation of menarcheal age to obesity in childhood and adulthood: The Bogalusa heart study. BMC Pediatr..

[24] He Q., Karlberg J. (2001). BMI in childhood and its association with height gain, timing of puberty, and final height. Pediatr. Res..

[25] Davison K.K., Susman E.J., Birch L.L. (2003). Percent body fat at age 5 predicts earlier pubertal development among girls at age 9. Pediatrics.

[26] Barlow S. (2007). Expert committee recommendations regarding the prevention, assessment and treatment of child and adolescent overweight and obesity: Summary report. Pediatrics.

[27] Daniels S.R., Arnett D.K., Eckel R.H., Gidding S.S., Hayman L.L., Kumanyika S., Robinson T.N., Scott B.J., St. Jeor S., Williams C.L. (2005). Overweight in children and adolescents: Pathophysiology, consequences, prevention, and treatment. Circulation.

[28] Moran A., Jacobs D.R., Steinberger J., Hong C.P., Prineas R., Luepker R., Sinaiko A.R. (1999). Insulin resistance during puberty: Results from clamp studies in 357 children. Diabetes.

[29] Goran M.I., Gower B.A. (2001). Longitudinal study on pubertal insulin resistance. Diabetes.

[30] Le Stunff C., Bougneres P. (1994). Early changes in postprandial insulin secretion, not in insulin sensitivity, characterize juvenile obesity. Diabetes.

[31] Amiel S.A., Sherwin R.S., Simonson D.C., Lauritano A.A., Tamborlane W.V. (1986). Impaired insulin action in puberty. A contributing factor to poor glycemic control in adolescents with diabetes. N. Engl. J. Med..

[32] Slyper A.H. (2006). The pubertal timing controversy in the USA, and a review of possible causative factors for the advance in timing of onset of puberty. Clin. Endocrinol..

[33] Triunfo S., Lanzone A. (2014). Impact of overweight and obesity on obstetric outcomes. J. Endocrinol. Invest..

[34] Sesé M.A., Jiménez-Pavón D., Gilbert C.C., González-Gross M., Gottrand F., de Henauw S., Breidenassel C., Wärnberg J., Widhalm K., Molnar D. (2012). Eating behaviour, insulin resistance and cluster of metabolic risk factors in European adolescents. The HELENA study. Appetite.

[35] Bringer J., Galtier F., Raingeard I., Boulot P., Renard E. (2008). Pregnancy and overweight: Underestimated consequences?. Bull. Acad. Natl. Med..

[36] National Health and Medical Research Council (2013). Clinical Practice Guidelines for the Management of Overweight and Obesity in Adults, Adolescents and Children in Australia.

[37] Graves L., Garnett S.P., Cowell C.T., Baur L.A., Ness A., Sattar N., Lawlor D.A. (2013). Waist-to-height ratio and cardiometabolic risk factors in adolescence: Findings from a prospective birth cohort. Pediatr. Obes..

[38] Dietz W.H. (2004). Overweight in childhood and adolescence. N. Eng. J. Med..

[39] Gordon-Larsen P., Nelson M.C., Popkin B.M. (2004). Longitudinal physical activity and sedentary behavior trends: Adolescence to adulthood. Amer. J. Prev. Med..

[40] Iannotti R.J., Wang J. (2013). Patterns of physical activity, sedentary behavior, and diet in U.S. adolescents. J. Adolesc. Health.

[41] Pereira H.R., Bobbio T.G., Antonio M.A., Barros Filho Ade A. (2013). Childhood and adolescent obesity: How many extra calories are responsible for excess of weight?. Rev. Paul. Pediatr..

[42] Cohen D.A., Ghosh-Dastidar B., Conway T.L., Evenson K.R., Rodriguez D.A., Beckman R., Elder J.P., Pickrel J., Lytle L. (2013). Energy balance in adolescent girls: The trial of activity for adolescent girls cohort. Obesity.

[43] Hills A.P., Byrne N.M., Lindstrom R., Hill J.O. (2013). “Small changes” to diet and physical activity behaviors for weight management. Obes. Facts.

[44] Damms-Machado A., Weser G., Bischoff S.C. (2012). Micronutrient deficiency in obese subjects undergoing low calorie diet. Nutr. J..

[45] Taheri S., Lin L., Austin D., Young T., Mignot E. (2004). Short sleep duration is associated with reduced leptin, elevated ghrelin, and increased body mass index. PLoS Med..

[46] Coldwell S.E., Oswald T.K., Reed D.R. (2009). A marker of growth differs between adolescents with high *vs.* low sugar preference. Physiol. Behav..

[47] Verma P., Mittal S., Ghildiyal A., Chaudhary L., Mahajan K.K. (2007). Salt preference: Age and sex related variability. Indian J. Physiol. Pharmacol..

[48] McNeil J., Cameron J.D., Finlayson G., Blundell J.E., Doucet E. (2013). Greater overall olfactory performance, explicit wanting for high fat foods and lipid intake during the mid-luteal phase of the menstrual cycle. Physiol. Behav..

[49] Utter J., Scragg R., Schaaf D. (2006). Associations between television viewing and consumption of commonly advertised foods among New Zealand children and young adolescents. Public Health Nutr..

[50] Gustafson A., Wu Q., Spees C., Putnam N., Adams I., Harp D., Bush H., Taylor C. (2014). How adolescents and parents food shopping patterns and social interaction when shopping is associated with dietary outcomes in rural communities. J. Obes. Weight Loss Ther..

[51] Trivedi S., Burton A., Oden J. (2013). Management of pediatric obesity: A lifestyle modification approach. Indian J. Pediatr..

[52] Thornton L.E., Cameron A.J., McNaughton S.A., Waterlander W.E., Sodergren M., Svastisalee C., Blanchard L., Liese A.D., Battersby S., Carter M.A. (2013). Does the availability of snack foods in supermarkets vary internationally?. Int. J. Behav. Nutr. Phys. Act..

[53] National Health and Medical Research Council (2013). Australian Dietary Guidelines.

[54] Mond J., van den Berg P., Boutelle K., Hannan P., Neumark-Sztainer D. (2011). Obesity, body dissatisfaction, and emotional well-being in early and late adolescence: Findings from the project EAT study. J. Adolesc. Health.

[55] Berge J.M., MacLehose R., Loth K.A., Eisenberg M., Bucchianeri M.M., Neumark-Sztainer D. (2013). Parent conversations about healthful eating and weight: Associations with adolescent disordered eating behaviors. JAMA Pediatr..

[56] Lai S.K., Costigan S.A., Morgan P.J., Lubans D.R., Stodden D.F., Salmon J., Barnett L.M. (2014). Do school-based interventions focusing on physical activity, fitness, or fundamental movement skill competency produce a sustained impact in these outcomes in children and adolescents? A systematic review of follow-up studies. Sport. Med..

[57] Lubans D.R., Morgan P.J., Cliff D.P., Barnett L.M., Okely A.D. (2010). Fundamental movement skills in children and adolescents: Review of associated health benefits. Sports Med..

[58] Janssen I., LeBlanc A.G. (2010). Systematic review of the health benefits of physical activity and fitness in school-aged children and youth. Int. J. Behav. Nutr. Phys. Act..

[59] Biddle S., Asare M. (2011). Physical activity and mental health in children and adolescents: A review of reviews. Brit. J. Sport. Med..

[60] Anderson L.B. (2013). Physical activity and cardiovascular disease risk factors in children: What can be done?. Amer. J. Lifestyle Med..

[61] Dempsey P., Owen N., Biddle S., Dunstan D. (2014). Managing sedentary behavior to reduce the risk of diabetes and cardiovascular disease. Curr. Diab. Rep..

[62] Pearson N., Braithwaite R., Biddle S., van Sluijs E., Atkin A.J. (2014). Associations between sedentary behaviour and physical activity in children and adolescents: A meta-analysis. Obes. Rev..

[63] Biddle S., Braithwaite R., Pearson N. (2014). The effectiveness of interventions to increase physical activity among young girls: A meta-analysis. Prev. Med..

[64] Chen G., Ratcliffe J., Olds T., Magarey A., Jones M., Leslie E. (2014). BMI, health behaviors, and quality of life in children and adolescents: A school-based study. Pediatrics.

[65] Velasquez-Rodriguez C.M., Velasquez-Villa M., Gomez-Ocampo L., Bermudez-Cardona J. (2014). Abdominal obesity and low physical activity are associated with insulin resistance in overweight adolescents: A cross-sectional study. BMC Pediatr..

[66] Sisson S.B., Shay C.M., Camhi S.M., Short K.R., Whited T. (2013). Sitting and cardiometabolic risk factors in U.S. adolescents. J. Allied Health.

[67] Saunders T.J., Chaput J.P., Tremblay M.S. (2014). Sedentary behaviour as an emerging risk factor for cardiometabolic diseases in children and youth. Can. J. Diab..

[68] Busch V., Manders L.A., de Leeuw J.R. (2013). Screen time associated with health behaviors and outcomes in adolescents. Amer. J. Health Behav..

[69] Mandic S., Leon de la Barra S., Garcia Bengoechea E., Stevens E., Flaherty C., Moore A., Middlemiss M., Williams J., Skidmore P. (2014). Personal, social and environmental correlates of active transport to school among adolescents in Otago, New Zealand. J. Sci. Med. Sport.

[70] Butt J., Weinberg R.S., Breckon J.D., Claytor R.P. (2011). Adolescent physical activity participation and motivational determinants across gender, age, and race. J. Phys. Act. Health.

[71] Wetton A.R., Radley R., Jones A.R., Pearce M.S. (2013). What are the barriers which discourage 15–16 year-old girls from participating in team sports and how can we overcome them?. Biomed. Res. Int..

[72] Moreno-Murcia J.A., Hellin P., Gonzalez-Cutre D. (2011). Influence of perceived sport competence and body attractiveness on physical activity and other healthy lifestyle habits in adolescents. Span. J. Psychol..

[73] Brockman R., Jago R., Fox K.R., Thompson J.L., Cartwright K., Page A.S. (2009). “Get off the sofa and go and play”: Family and socioeconomic influences on the physical activity of 10–11 year old children. BMC Public Health.

[74] Taylor S.J., Barker L.A., Heavey L., McHale S. (2013). The typical developmental trajectory of social and executive functions in late adolescence and early adulthood. Dev. Psychol..

[75] Liang J., Matheson B.E., Kaye W.H., Boutelle K.N. (2014). Neurocognitive correlates of obesity and obesity-related behaiors in children and adolescents. Int. J. Obes..

[76] Maayan L., Hoogendoorn C., Sweat V., Convit A. (2011). Disinhibited eating in obese adolescents is associated with orbitofrontal volume reductions and executive dysfunction. Obesity.

[77] Evans G.W., Fuller-Rowell T.E., Doan S.N. (2012). Childhood cumulative risk and obesity: The mediating role of self-regulatory ability. Pediatrics.

[78] Wonderlich S.A., Connolly K.M., Stice E. (2004). Impulsivity as a risk factor for eating disorder behavior: Assessment implications with adolescents. Int. J. Eat. Disord..

[79] Morgan A.Z., Keiley M.K., Ryan A.E., Radomski J.G., Gropper S.S., Connell L.J., Simmons K.P., Ulrich P.V. (2012). Eating regulation styles, appearance schemas, and body satisfaction predict changes in body fat for emerging adults. J. Youth Adolesc..

[80] Diamond A., Lee K. (2011). Interventions shown to aid executive function development in children 4 to 12 years old. Science.

[81] Fuemmeler B.F., Yang C.M., Costanzo P., Hoyle R.H., Siegler I.C., Williams R.B., Ostbye T. (2012). Parenting styles and body mass index trajectories from adolescence to adulthood. Health Psychol..

[82] Phillips T.M. (2008). Age-related difference in identity style: A cross-sectional sample. Curr. Psycol..

[83] Burke P.J. (2006). Identity change. Soc. Psychol. Quart..

[84] Strachan S.M., Woodgate J., Brawley L.R., Tse A. (2005). The relationship of self efficacy and self identity to long term maintenance of vigorous physical activity. J. Appl. Biobehav. Res..

[85] Strachan S.M., Brawley L.R. (2009). Healthy-eater identity and self-efficacy predict healthy eating behavior: A prospective view. J. Health Psychol..

[86] Krug I., Villarejo C., Jimenez-Murcia S., Perpina C., Vilarrasa N., Granero R., Cebolla A., Botella C., de Bernabe M.M.G., Penelo E. (2013). Eating-related Environmental Factors in underweight eating disorders and obesity: Are there common vulnerabilities during childhood and early adolescence?. Eur. Eat. Disord. Rev..

[87] Dickens E., Ogden J. (2014). The role of parental control and modelling in predicting a childʼs diet and relationship with food after they leave home. A prospective study. Appetite.

[88] Gillison F., Standage M., Verplanken B. (2014). A cluster randomised controlled trial of an intervention to promote healthy lifestyle habits to school leavers: Study rationale, design, and methods. BMC Public Health.

[89] De Bruijn G.J., van den Putte B. (2012). Exercise promotion: An integration of exercise self-identity, beliefs, intention, and behaviour. Eur. J. Sport Sci..

[90] Okely A.D., Cotton W.G., Lubans D.R., Morgan P.J., Puglisi L., Miller J., Wright J., Batterham M.J., Peralta L.R., Perry J. (2011). A school-based intervention to promote physical activity among adolescent girls: Rationale, design, and baseline data from the girls in sport group randomised controlled trial. BMC Public Health.

[91] Singh A.S., Chin A.P.M.J., Kremers S.P., Visscher T.L., Brug J., van Mechelen W. (2006). Design of the Dutch Obesity Intervention in Teenagers (NRG-DOiT): Systematic development, implementation and evaluation of a school-based intervention aimed at the prevention of excessive weight gain in adolescents. BMC Public Health.

[92] Sui Z., Turnbull D.A., Dodd J.M. (2012). Overweight and obese women’s perceptions about making healthy change during pregnancy: A mixed method study. Matern. Child. Health J..

[93] Kalavainen M.P., Korppi M.O., Nuutinen O.M. (2007). Clinical efficacy of group-based treatment for childhood obesity compared with routinely given individual counseling. Int. J. Obes..

[94] Okely A.D., Collins C.E., Morgan P.J., Jones R.A., Warren J.M., Cliff D.P., Burrows T.L., Colyvas K., Steele J.R., Baur L.A. (2010). Multi-site randomized controlled trial of a child-centered physical activity program, a parent-centered dietary-modification program, or both in overweight children: The HIKCUPS study. J. Pediatr..

[95] Kelly S.A., Melnyk B.M. (2008). Systematic review of multicomponent interventions with overweight middle adolescents: Implications for clinical practice and research. Worldviews Evid. Based Nurs..

[96] Utter J., Denny S., Dixon R., Ameratunga S., Teevale T. (2013). Family support and weight-loss strategies among adolescents reporting sustained weight loss. Public Health Nutr..

[97] Larson N.I., Story M., Eisenberg M.E., Neumark-Sztainer D. (2006). Food preparation and purchasing roles among adolescents: Associations with sociodemographic characteristics and diet quality. J. Amer. Diet. Assn..

[98] Utter J., Denny S., Robinson E., Fleming T., Ameratunga S., Grant S. (2013). Family meals and the well-being of adolescents. J. Paediatr. Child. Health.

[99] Sato A.F., Jelalian E., Hart C.N., Lloyd-Richardson E.E., Mehlenbeck R.S., Neill M., Wing R.R. (2011). Associations between parent behavior and adolescent weight control. J. Pediatr. Psychol..

[100] King L.A., Loss J.H., Wilkenfeld R.L., Pagnini D.L., Booth M.L., Booth S.L. (2007). Australian GPSʼ perceptions about child and adolescent overweight and obesity: The weight of opinion study. Brit. J. Gen. Pract..

[101] Shrewsbury V., King L., Hattersley L., Howlett S., Hardy L., Baur L. (2010). Adolescent-parent interactions and communication preferences regarding body weight and weight management: A qualitative study. Int. J. Behav. Nutr. Phys. Act..

[102] Moy J., Petrie T.A., Dockendorff S., Greenleaf C., Martin S. (2013). Dieting, exercise, and intuitive eating among early adolescents. Eat. Behav..

[103] Cook T., Rutishauser I., Seelig M. (2001). Comparable Data on Food and Nutrient Intake and Physical Measurements from the 1983, 1985 and 1995 National Nutrition Surveys.

[104] (2013). Measured Obesity in Queensland 2011–2012.

[105] (2011). A Modelling System to Inform the Revision of the Australian Guide to Healthy Eating.

[106] Buckland G., Bach A., Serra-Majem L. (2008). Obesity and the Mediterranean diet: A systematic review of observational and intervention studies. Obes. Rev..

[107] Steenhuis I.H., Vermeer W.M. (2009). Portion size: Review and framework for interventions. Int. J. Behav. Nutr. Phys. Act..

[108] Leidy H.J., Ortinau L.C., Douglas S.M., Hoertel H.A. (2013). Beneficial effects of a higher-protein breakfast on the appetitive, hormonal, and neural signals controlling energy intake regulation in overweight/obese, “breakfast-skipping”, late-adolescent girls. Amer. J. Clin. Nutr..

[109] Dietz W.H. (1997). Periods of risk in childhood for the development of adult obesity—What do we need to learn?. J. Nutr..

[110] Ho M., Garnett S.P., Baur L.A., Burrows T., Stewart L., Neve M., Collins C. (2013). Impact of dietary and exercise interventions on weight change and metabolic outcomes in obese children and adolescents: A systematic review and meta-analysis of randomized trials. JAMA Pediatr..

[111] Larson N., MacLehose R., Fulkerson J.A., Berge J.M., Story M., Neumark-Sztainer D. (2013). Eating breakfast and dinner together as a family: Associations with sociodemographic characteristics and implications for diet quality and weight status. J. Acad. Nutr. Diet..

[112] Abrams P., Levitt Katz L.E., Moore R.H., Xanthopoulos M.S., Bishop-Gilyard C.T., Wadden T.A., Berkowitz R.I. (2013). Threshold for improvement in insulin sensitivity with adolescent weight loss. J. Pediatr..

[113] Olshansky S.J., Passaro D.J., Hershow R.C., Layden J., Carnes B.A., Brody J., Hayflick L., Butler R.N., Allison D.B., Ludwig D.S. (2005). A potential decline in life expectancy in the United States in the 21st Century. N. Eng. J. Med..

[114] Hills A., Okely A., Baur L. (2010). Addressing childhood obesity through increased physical activity. Nat. Rev. Endocrinol..

[115] Andersen L.B., Riddoch C., Kriemler S., Hills A.P. (2011). Physical activity and cardiovascular risk factors in children. Brit. J. Sport. Med..

[116] Telama R., Yang X., Leskinen E., Kankaanpää A., Hirvensalo M., Tammelin T., Viikari J.S., Raitakari O.T. (2014). Tracking of physical activity from early childhood through youth into adulthood. Med. Sci. Sports Exerc..

[117] Pearson N., Braithwaite R., Biddle S.J.H. (2015). The effectiveness of interventions to increase physical activity among adolescent girls: A meta-analysis. Acad. Pediatr..

[118] Martinez-Vizcaino V., Sanchez-Lopez M., Notario-Pacheco B., Salcedo-Aguilar F., Solera-Martinez M., Franquelo-Morales P., Lopez-Martinez S., Garcia-Prieto J.C., Arias-Palencia N., Torrijos-Nino C. (2014). Gender differences on effectiveness of a school-based physical activity intervention for reducing cardiometabolic risk: A cluster randomized trial. Int. J. Behav. Nutr. Phys. Act..

[119] Duncan S.C., Strycker L.A., Chaumeton N.R. (2015). School influences on the physical activity of african american, latino, and white girls. J. Sch. Health.

[120] Haapala H.L., Hirvensalo M.H., Laine K., Laakso L., Hakonen H., Kankaanpaa A., Lintunen T., Tammelin T.H. (2014). Recess physical activity and school-related social factors in Finnish primary and lower secondary schools: Cross-sectional associations. BMC Public Health.

[121] Lopes A.A., Lanzoni A.N., Hino A.A., Rodriguez-Anez C.R., Reis R.S. (2014). Perceived neighborhood environment and physical activity among high school students from Curitiba, Brazil. Rev. Bras. Epidemiol..

[122] Mitchell J.A., Rodriguez D., Schmitz K.H., Audrain-McGovern J. (2013). Sleep duration and adolescent obesity. Pediatrics.

[123] Wren A.M., Seal L.J., Cohen M.A., Brynes A.E., Frost G.S., Murphy K.G., Dhillo W.S., Ghatei M.A., Bloom S.R. (2001). Ghrelin enhances appetite and increases food intake in humans. J. Clin. Endocrinol. Metab..

[124] Schmid S.M., Hallschmid M., Lehnert H., Schultes B. (2010). Short-term sleep loss decreases physical activity under free-living conditions but does not increase food intake under time-deprived laboratory conditions in healthy men. Reply to J-P Chaput *et al*. Amer. J. Clin. Nutr..

[125] Maggard M.A., Yermilov I., Li Z., Maglione M., Newberry S., Suttorp M., Hilton L., Santry H.P., Morton J.M., Livingston E.H. (2008). Pregnancy and fertility following bariatric surgery: A systematic review. J. Amer. Med. Assn..

[126] Sarrafzadegan N., Rabiei K., Wong F., Roohafza H., Zarfeshani S., Noori F., Grainger-Gasser A. (2014). The sustainability of interventions of a community-based trial on children and adolescents’ healthy lifestyle. ARYA Atheroscler..

[127] Herring S.J., Rose M.Z., Skouteris H., Oken E. (2012). Optimizing weight gain in pregnancy to prevent obesity in women and children. Diab. Obes. Metab..

[128] Logsdon M.C., Bennett G., Crutzen R., Martin L., Eckert D., Robertson A., Myers J., Tomasulo R., Gregg J., Barone M. (2014). Preferred health resources and use of social media to obtain health and depression information by adolescent mothers. J. Child. Adolesc. Psychiatr. Nurs..

[129] Bottorff J.L., Struik L.L., Bissell L.J., Graham R., Stevens J., Richardson C.G. (2014). A social media approach to inform youth about breast cancer and smoking: An exploratory descriptive study. Collegian.

[130] Jones K., Eathington P., Baldwin K., Sipsma H. (2014). The impact of health education transmitted via social media or text messaging on adolescent and young adult risky sexual behavior: A systematic review of the literature. Sex Transm. Dis..

